# A cocktail of three virulent bacteriophages prevents *Vibrio cholerae* infection in animal models

**DOI:** 10.1038/ncomms14187

**Published:** 2017-02-01

**Authors:** Minmin Yen, Lynne S. Cairns, Andrew Camilli

**Affiliations:** 1 Department of Molecular Biology and Microbiology, Howard Hughes Medical Institute, Tufts University School of Medicine, 136 Harrison Avenue, Boston, Massachussetts 02111, USA; 2 Program in Molecular Microbiology, Sackler School of Graduate Biomedical Sciences, Tufts University, Boston, Massachussetts 02111, USA

## Abstract

Effective prevention strategies will be essential in reducing disease burden due to bacterial infections. Here we harness the specificity and rapid-acting properties of bacteriophages as a potential prophylaxis therapy for cholera, a severely dehydrating disease caused by *Vibrio cholerae*. To this end, we test a cocktail of three virulent phages in two animal models of cholera pathogenesis (infant mouse and rabbit models). Oral administration of the phages up to 24 h before *V. cholerae* challenge reduces colonization of the intestinal tract and prevents cholera-like diarrhea. None of the surviving *V. cholerae* colonies are resistant to all three phages. Genome sequencing and variant analysis of the surviving colonies indicate that resistance to the phages is largely conferred by mutations in genes required for the production of the phage receptors. For acute infections, such as cholera, phage prophylaxis could provide a strategy to limit the impact of bacterial disease on human health.

## Abstract (web summary)

There has been a renewed interest in the potential use of bacterial viruses (phages) for treatment or prevention of bacterial infections. Here the authors show that a cocktail of three phages can prevent cholera-like diarrhoea in animals infected with *Vibrio cholerae*.

Cholera is an acute, severely dehydrating diarrheal disease caused by the water-borne bacterium *Vibrio cholerae.* Cholera remains a substantial global health burden and is endemic to many parts of Africa and Asia^1^. Recent widespread epidemics in disaster-stricken or war-torn countries such as Haiti^2^ and Iraq^3^ highlight the vulnerabilities of populations to sudden outbreaks. Current recommended preventatives include mass vaccinations with the World Health Organization-prequalified oral cholera vaccine^4^ and increased awareness of sanitation and hygiene practices^5^. However, access to clean water is difficult and vaccination campaigns require forethought and time for efficacy; both methods may not be logistically feasible for immediate protection in the event of an outbreak. Household transmission is a major contributor to the rapid spread of *V. cholerae* within communities. Household contacts of index cases often present with cholera symptoms 2–3 days after the initial patient becomes sick^6^. Therefore, there is currently an unmet need for a clinical intervention to stem the household spread of cholera by use of a rapid prophylactic treatment. Although chemoprophylaxis with antibiotics may effectively reduce cholera burden^7^, the World Health Organization does not recommend this practice due to the development and spread of drug-resistant bacteria ( http://www.who.int/cholera/technical/prevention/control/en/). Moreover, the broad-spectrum action of antibiotics would cause dysbiosis of the resident intestinal microbiota, which could put patients at risk of other intestinal infections.

There has recently been a renewed interest in the use of bacteriophages (phages) for environmental and clinical applications^8^. In contrast to antibiotics, phages are specific in their targets and, because they are replicating viruses, are capable of auto-dosing, a phenomenon where phage replication increases their number and contributes to the dose. Previous attempts to use phages to prevent or treat cholera have produced mixed results. Dutta *et al*.^9^ showed that a single phage type given 1 h before *V. cholerae* challenge in an infant rabbit model prevented onset of cholera symptoms. Studies by Jaiswal *et al*.^10^ showed that a cocktail of five lytic bacteriophage types given 6 or 12 h before *V. cholerae* challenge in an adult rabbit model reduced diarrheal severity slightly but did not significantly lower the bacterial load; however, the same phage cocktail could successfully reduce the *V. cholerae* load when administered 6 or 12 h after challenge. A second study in adult mice also showed promise for treatment of cholera with a phage cocktail^11^. These studies suggest that a prophylaxis approach merits further, in-depth investigation.

We previously isolated three *V. cholerae*-specific, lytic (virulent) phages ICP1, ICP2 and ICP3 from rice-water stool samples of cholera patients in Bangladesh^12^. The receptors for ICP1 and ICP2 were identified as the lipopolysaccharide (LPS) O1 antigen^13^ and the major outer membrane porin OmpU^14^, respectively, which are considered virulence factors of *V. cholerae*. The receptor for ICP3 is as yet unknown, although we hypothesize at least a partial role for the LPS O-antigen. We have recently shown that these virulent phages impose significant bactericidal pressure on *V. cholerae* during its natural course of infection in humans^14^. A cocktail comprising phages that target different receptors would reduce the likelihood of multi-phage-resistant *V. cholerae* isolates in the surviving population. Therefore, we hypothesized that a cocktail of the three ICP phages may be used as a prophylaxis treatment to specifically target *V. cholerae* that transits into the small intestine to prevent signs of cholera in animal models of disease.

In this study, we show that orally applied, prophylactic use of the ICP cocktail reduces colonization by *V. cholerae* in the infant mouse model. The ICP cocktail also prevents the onset of cholera symptoms in the infant rabbit model when administered up to 24 h before *V. cholerae* challenge. This proof-of-principle study demonstrates the successful use of phage prophylaxis to prevent disease caused by a mucosal pathogen.

## Results

### The three-phage ICP cocktail kills *V. cholerae* *in vitro*

**Figure 1:**
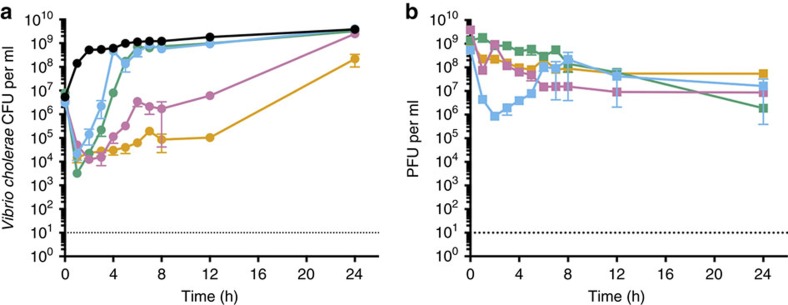
The ICP cocktail kills *V. cholerae in vitro.* Growth curves of *V. cholerae* E7946 (AC53) in the absence and presence of phage predation. Cultures were grown in the absence of phage (black) or with ICP1 (blue), ICP2 (pink), ICP3 (green) or the ICP phage cocktail (orange) at an multiplicity of infection of 1. Samples of each culture were taken periodically and (**a**) enumerated for CFU per ml or (**b**) the phage fraction isolated and enumerated for PFU per ml by plaque assay. Data are plotted as the average of five independent replicates. Error bars represent the s.e.m. The dotted line represents the limit of detection.

Initial *in vitro* killing time-course experiments provided support for the hypothesis that the three-phage ICP cocktail was more effective in killing *V. cholerae* than each phage in isolation (Fig. 1). Cultures of *V. cholerae* E7946 (AC53) were grown in (i) the absence of phage or in the presence of (ii) ICP1, (iii) ICP2, (iv) ICP3 or (v) the ICP cocktail, at a multiplicity of infection of 1. Bacteria (Fig. 1a) and phage (Fig. 1b) titres were enumerated every hour for 8 h and then again at 12 and 24 h. Although the bacterial population declined in all conditions initially, growth resumed for cultures grown in the presence of ICP1 or ICP3 within 4–6 h, respectively (Fig. 1a). Cells incubated in the presence of ICP2 resumed growth more slowly, but reached the same density as the control within 24 h. In contrast, cells grown with the ICP cocktail did not reach the same density as the no-phage control by the end of the experiment. Phage titres dropped but remained stable for all conditions over the course of the experiment (Fig. 1b). It is not surprising that *V. cholerae* was able to escape phage predation over time, given that the starting inoculum was high (5 × 10^7^ colony-forming units (CFU)) and probably contained phage-resistant mutants that were positively selected.

### The ICP cocktail reduces mouse small intestinal colonization

**Figure 2:**
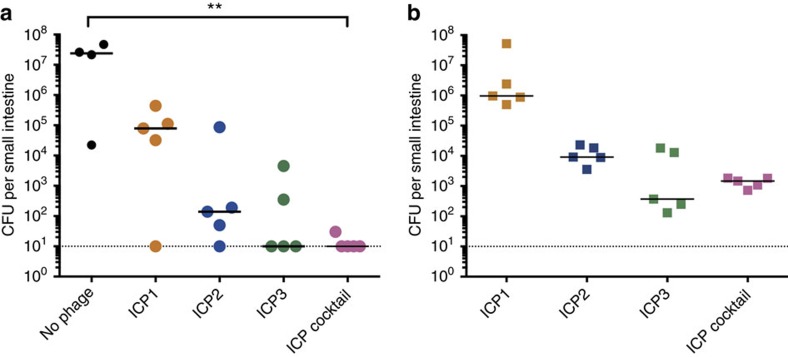
Efficacy of the ICP cocktail and the ICP phages in reducing *V. cholerae* colonization of the infant mouse small intestine. Mice were dosed with between 1 × 10^6^ and 1 × 10^7^ PFU of the indicated phages 3 h before infection with 5 × 10^5^ CFU *V. cholerae* E7946 (AC53). A control group did not receive phage (*n*=4). For each phage-treated group of animals, *n*=5. (**a**) *V. cholerae* that survived in the small intestine were enumerated as CFU per small intestine. (**b**) Phages that survived in the small intestine were enumerated as PFU per small intestine by plaque assay. The dotted line represents the limit of detection and the horizontal solid bar represents the median. Each square or circle represents one animal. Significance was calculated using the Kruskal–Wallis test with the Dunn's *post-hoc* multiple comparisons test. **P=0.001–0.01.

The ICP cocktail was more effective than each phage alone in killing *V. cholerae in vitro*. To test whether this was also true *in vivo*, prophylaxis experiments were performed in the infant mouse model of *V. cholerae* colonization. We hypothesized that the ICP cocktail would be effective in preventing *V. cholerae* infection of the infant mouse small intestine. Mice were divided into five groups and received either (i) no phage, (ii) ICP1, (iii) ICP2, (iv) ICP3 or (v) the ICP cocktail. Mice were given phages (between 1 × 10^6^ and 1 × 10^7^ plaque-forming units (PFU)) by orogastric inoculation 3 h before infection with 5 × 10^5^ CFU *V. cholerae*. After 24 h, mice were killed, and *V. cholerae* and phages in the small intestine enumerated. As shown in Fig. 2a, the number of surviving *V. cholerae* cells in the small intestine was reduced by at least two orders of magnitude in all conditions where phage was administered, in comparison with the non-treated control, with ICP3 being the most successful single phage. No *V. cholerae* were detected in the intestinal homogenates of four out of five mice treated with the ICP cocktail (*P*<0.01, Kruskal–Wallis test with the Dunn's *post-hoc* multiple comparisons test). These data show that in comparison with ICP1 or ICP2 alone, ICP3 alone or the ICP cocktail is superior at preventing *V. cholerae* colonization. Phages were still detected in the small intestine at the end of the experiment (Fig. 2b), suggesting that these phages can survive and persist in the intestinal tract. Although the bacterial count medians of the ICP3 alone and the ICP cocktail groups are similar, we chose to proceed with the ICP cocktail to reduce the probability that phage-resistant *V. cholerae* isolates would survive.

**Figure 3:**
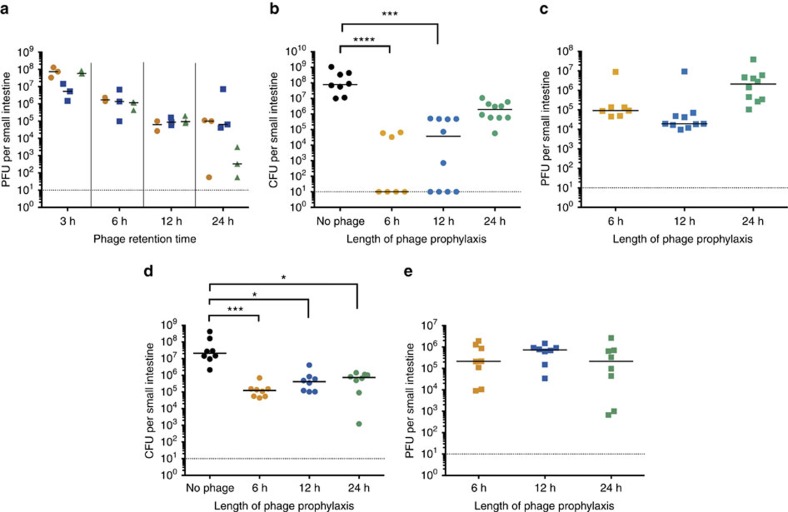
*V. cholerae* burden in the infant mouse small intestine is reduced when the ICP cocktail is administered up to 24 h before infection. (**a**) Phages are retained in the infant mouse small intestine in the absence of the *V. cholerae* host. Mice were dosed with between 3 × 10^7^ and 3 × 10^8^ PFU of the indicated phage and killed after 3, 6, 12 or 24 h. Phages surviving in the small intestine were calculated by enumeration of PFU per small intestine from plaque assays. An orange circle represents ICP1, a blue square ICP2 and a green triangle ICP3. For each group, *n*=3. (**b**) Infant mice were dosed with between 2 × 10^5^ and 4.8 × 10^5^ PFU of the ICP cocktail either 6 h (*n*=7), 12 h (*n*=10) or 24 h (*n*=10) before infection with between 5 × 10^5^ and 9 × 10^5^ CFU *V. cholerae* E7946 (AC53). A control group did not receive phage (*n*=8). Twenty-four hours after infection, the mice were killed and *V. cholerae* surviving in the small intestine enumerated by calculation of CFU per small intestine. (**c**) Phages surviving in the small intestine after the challenge described in **b** were enumerated by calculation of PFU per small intestine from plaque assays. (**d**) Infant mice were dosed with between 1 × 10^8^ and 1.3 × 10^8^ PFU of the ICP cocktail 6 h (*n*=8), 12 h (*n*=8) or 24 h (*n*=8) before infection with a ‘high-challenge dose' of 1 × 10^8^ CFU *V. cholerae* E7946 (AC53). Twenty-four hours after infection, mice were killed and the *V. cholerae* surviving in the small intestine enumerated as CFU per small intestine, and phages enumerated as PFU per small intestine by plaque assay (**e**). The dotted line represents the limit of detection and the horizontal solid bar represents the median. Each square or circle represents one animal. Significance was calculated using the Kruskal–Wallis test with the Dunn's *post-hoc* multiple comparisons test. *P=0.01–0.05, ***P=0.0001–0.001 and ****P<0.0001.

Next, we aimed to test whether the ICP cocktail could still be effective when administered up to 24 h before *V. cholerae* infection. First, it was necessary to establish if phages could survive in the small intestine for long periods in the absence of their host bacterium. To do this, mice were dosed with phages (between 3 × 10^7^ and 3 × 10^8^ PFU) by oral gavage and killed after 3, 6, 12 or 24 h. Phages were enumerated from intestinal homogenates using plaque assays (Fig. 3a). After 3 h, the phage titres for ICP1 and ICP3 were still stable, whereas ICP2 titres had dropped ∼10-fold. For all three phages, titres dropped only 10- to 100-fold after 6–12 h. After 24 h, ICP1 and ICP2 were still retained at ∼10^5^ PFU per small intestine, whereas the titre of ICP3 fell to between 10^2^ and 10^4^ PFU per small intestine. These data further show that the ICP phages can survive in the intestinal tract for at least 24 h in the absence of their host *V. cholerae*.

To test whether the ICP cocktail remained protective when given several hours before exposure to *V. cholerae*, infant mice were inoculated with the ICP cocktail 6, 12 or 24 h before challenge with between 5 × 10^5^ and 9 × 10^5^ CFU *V. cholerae.* A control group did not receive phage. The data presented in Fig. 3b show that the 6 h prophylaxis was most successful; the number of surviving *V. cholerae* cells fell at least three orders of magnitude, in comparison with the no-phage group, with no detectable *V. cholerae* for four of the seven mice that received phage (*P*<0.0001, Kruskal–Wallis test with the Dunn's *post-hoc* multiple comparisons test). The number of *V. cholerae* cells was also significantly lower for the 12 h group (*P*<0.001), with four of these animals having no detectable *V. cholerae* at the end of the experiment. The number of *V. cholerae* cells was also reduced two orders of magnitude in the 24 h group. Furthermore, virulent phages were still detectable 24 h post-infection (Fig. 3c).

To determine whether the ICP cocktail could limit colonization when mice are given a higher challenge dose, infant mice were inoculated with 1 × 10^8^ CFU of *V. cholerae*, a dose ∼200-fold higher than the experiment shown in Fig. 3b,c. The data presented in Fig. 3d show that the ICP cocktail was effective in reducing *V. cholerae* colonization of the small intestine by at least two orders of magnitude when administered at either 6, 12 or 24 h before challenge. Phages were also still detected at 24 h post infection at similar levels in all three phage-treated groups (Fig. 3e). In concert, these data support the hypothesis that a phage prophylaxis approach can be used to prevent *V. cholerae* colonization of the small intestine.

*V. cholerae* could be isolated from intestinal homogenates of several of the mice dosed with the ICP cocktail (Figs 2 and 3). Owing to the complexity of infection within the gut, it is possible that these bacteria survive because either they did not encounter the ICP phages or these cells carry genetic mutations that allow them to escape phage predation. To determine the phage resistance profile of surviving cells, *V. cholerae* isolates from the experiment in Fig. 3b,c were randomly selected for colony purification and used in efficiency of plating (EOP) assays. The data are summarized in Supplementary Table 1 and detailed in Supplementary Data 1. All isolates from mice that received the ICP cocktail 6 or 12 h before challenge were sensitive to all three ICP phages. The majority of isolates from mice given the ICP cocktail 24 h before challenge were sensitive to all three ICP phages; however, a small number showed differing ICP resistance phenotypes.

To uncover the genetic basis for resistance, 24 *V. cholerae* isolates were analysed by whole-genome sequencing followed by variant analysis^15^,^16^,^17^. It was shown previously that slipped-strand mispairing in the poly-A tracts of O-antigen synthesis genes can result in abnormal O-antigen and confer ICP1 resistance^13^. Consistent with this, the mutations in ICP1- and ICP3-resistant isolates were found in O-antigen synthesis genes located on chromosome 1 of *V. cholerae* between open reading frames VC0240 (*gmhD*) and VC0269 (*manA*)^18^ (Supplementary Data 2). Although LPS mutations were a common source of resistance in these isolates, this is not of major concern given that strains carrying such mutations have previously been shown to be avirulent^19^,^20^,^21^. We have also shown previously that mutations in *ompU* and *toxR* confer ICP2 resistance^14^. In agreement, mutations in ICP2-resistant isolates were found in open reading frames VC0633 (*ompU*) or VC0984 (*toxR*) (Supplementary Data 2). Eight of the sequenced strains revealed no mutations to explain their phage-resistant phenotypes, most likely due to incomplete genome sequence coverage.

### The ICP cocktail provides protection in the infant rabbit

**Figure 4:**
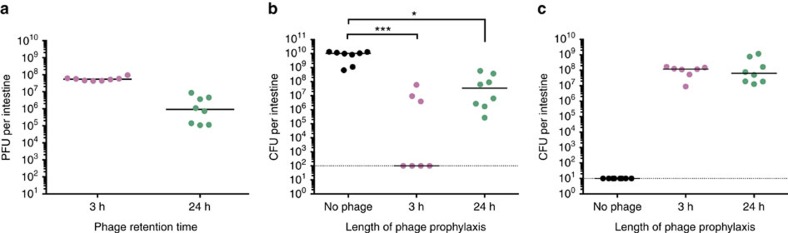
Infant rabbits dosed with the ICP phage cocktail before *V. cholerae* infection have a reduced bacterial load in the intestine. (**a**) Infant rabbits were dosed with 3 × 10^8^ PFU of the ICP phage cocktail and killed after 3 h (*n*=8) or 24 h (*n*=8). Phage retention in the intestine in the absence of the *V. cholerae* host was measured by enumeration of PFU per intestine. (**b**) Infant rabbits were dosed with between 4 × 10^9^ and 8 × 10^9^ PFU of the ICP phage cocktail either 3 h (*n*=7) or 24 h (*n*=8) before infection with 5 × 10^8^ CFU *V. cholerae* E7946 (AC53). A control group (*n*=8) did not receive phage. At the end of the infection period (either 12–14 h for the *V. cholerae* only group, or 20 h after *V. cholerae* infection for the phage-treated groups (see Methods for more details)), rabbits were killed and *V. cholerae* surviving in the intestine were enumerated as CFU per intestine. (**c**) Phages surviving in the intestine after the challenge described in **b** were enumerated as PFU per intestine. The dotted line represents the limit of detection and the horizontal solid bar represents the median. Each square or circle represents one animal. Significance was calculated using the Kruskal–Wallis test with the Dunn's *post-hoc* multiple comparisons test. *P=0.01–0.05 and ***P=0.0001–0.001.

To further assess the ability of the ICP cocktail to prevent cholera, experiments were undertaken in infant rabbits. In contrast to infant mice, infant rabbits infected by *V. cholerae* develop the profuse secretory diarrhea associated with cholera. Sickened animals suffer from dehydration and weight loss and show an accumulation of caecal fluid containing a high bacterial load^22^. First, to establish whether phages could be retained in the rabbit intestinal tract in the absence of *V. cholerae*, animals were dosed with the ICP cocktail alone (3 × 10^8^ PFU). After 3 or 24 h, the animals were killed and phages enumerated from intestinal homogenates. Even after 24 h, 10^6^ phages could be recovered from the intestine (Fig. 4a).

To determine whether the ICP cocktail could block infection by *V. cholerae*, phages (between 4 × 10^9^ and 8 × 10^9^ PFU) were orogastrically administered to two groups of infant rabbits either 3 or 24 h before challenge with 5 × 10^8^ CFU *V. cholerae*. A control group did not receive phage. Animals were monitored for signs of cholera, specifically weight loss (Supplementary Fig. 1) and the presence of rice-water stool (also referred to here as cholera-like diarrhea). The no-phage control group showed cholera-like symptoms 12–14 h post infection, in line with previously published observations^22^, and were killed at this point. The caecum of each infected rabbit was distended and accumulated ∼0.5–1 ml of fluid, which is further indicative of *V. cholerae* infection. Approximately 10^9^–10^10^ CFU were enumerated from caecal fluid (Supplementary Table 2) and intestinal homogenates (Fig. 4b) from this group.

*V. cholerae* were not detected for four of the seven rabbits that were dosed with ICP cocktail 3 h before infection (Fig. 4b), indicating that these animals cleared the infection. Between 10^6^ and 10^8^ CFU of *V. cholerae* were obtained from intestinal homogenates from the remaining three rabbits in this group, giving a 10- to 100,000-fold decrease in comparison with the non-treated group (Fig. 4b, *P*<0.001, Kruskal–Wallis test with the Dunn's *post-hoc* multiple comparisons test). Between ∼10^5^ and 10^9^ CFU of *V. cholerae* were enumerated from the intestines of animals dosed with phage 24 h before infection (Fig. 4b), presenting a 10- to 100,000-fold decrease in bacterial load when compared with animals that received no phage (*P*<0.05). It is important to note that despite the bacterial load observed, there was no evidence of rice-water stool or significant weight loss for rabbits in either of the phage-dosed groups up to 20 h after *V. cholerae* challenge (Supplementary Fig. 1). On dissection of these animals, 0.1 ml of caecal fluid was obtained from one rabbit, which was dosed with phage 24 h before *V. cholerae* infection. The caeca of the other animals showed no signs of fluid accumulation (Supplementary Table 2). Between 10^6^ and 10^9^ PFU could be collected from the intestinal homogenates of phage-treated animals (Fig. 4c), indicating that phages persisted in the intestine over the course of the experiment. These observations suggest that the ICP cocktail protected against the signs of cholera over the duration of the experiment.

To determine whether *V. cholerae* that survived phage prophylaxis were sensitive or resistant to the ICP phages, *V. cholerae* isolates from each of the intestinal homogenates were randomly selected for colony purification and their phage resistance phenotype measured by EOP assays (summarized in Supplementary Table 3 and detailed in Supplementary Data 3). Thirty-nine per cent of isolates were resistant to both ICP1 and ICP3. No isolates were resistant to all three phages. To determine the genetic basis for resistance, whole-genome sequencing and variant analysis were conducted on 36 isolates, chosen due to their differing resistance phenotypes (Supplementary Data 4). Results were consistent with mutations found in ICP1- and ICP3-resistant isolates from the mouse experiment in Fig. 3. For 33 of these strains, there were mutations in O-antigen synthesis genes. For three strains, no mutations related to known phage-resistance strategies were detected. We conclude from these data in two animal models that the leading source of phage resistance is mutation of the genes required for production of the phage receptors.

Collectively, these data suggest that the phage cocktail (i) is efficient at killing *V. cholerae* cells *in vivo*, (ii) reduces or prevents *V. cholerae* colonization of the intestine and (iii) provides protection against the onset of cholera-like diarrhoea in the infant rabbit model of cholera infection. Furthermore, isolates shed by the animal that were resistant to the ICP phages are expected to be avirulent based on past studies of O1-antigen mutants^13^, although virulence of these isolates has not yet been tested.

## Discussion

Here we show that prophylactic administration of a *V. cholerae*-specific phage cocktail is protective against cholera, by reducing both colonization and cholera-like diarrhoea in two animal models. We have shown that the ICP cocktail can successfully kill *V. cholerae in vitro*, prevent colonization of the infant mouse and preclude the onset of cholera-like diarrhoea in the infant rabbit. *V. cholerae* were isolated from intestinal homogenates of some phage-treated animals; however, the surviving population is smaller than observed from a wild-type *V. cholerae* infection (Figs 2, 3, 4). Moreover, we hypothesize that these surviving isolates may be unable to elicit acute infection, as many of the mutations that confer resistance to the ICP phages affect genes such as those involved in LPS synthesis (VC0240 to VC0269), which have been previously shown to be important for virulence. Crucially, at the end of these experiments, the population also remains sensitive to at least one of the three ICP phages. We suggest that the effectiveness of the ICP cocktail in killing *V. cholerae* cells is at least in part reliant on the concentration of phages that are in the intestine at the time of *V. cholerae* infection. The complex architecture of the intestinal tract probably presents a challenge to the phages' ability to access *V. cholerae* cells. The details of the interplay between the ICP phages, *V. cholerae* and the host remain to be elucidated.

Future optimization of the composition and dose of the cocktail could further improve its effectiveness. The ratio of each phage in the cocktail could be optimized and additional phages could be added to improve *V. cholerae* killing. Furthermore, as complete reduction of the bacterial load was only observed at shorter prophylaxis times, optimization of the timing for each dose is needed before human use. It is likely that an individual will be repeatedly exposed to *V. cholerae*; therefore multiple doses may be necessary to control the infection to a point where it can be completely eradicated.

Current phage therapy research is largely focused on treating ongoing infections. Our results highlight the potential of phage therapy in preventing infections. The data presented herein suggest that the premise of using a phage cocktail to prevent cholera warrants further investigation. We anticipate that a rapid-acting phage prophylaxis approach would be best used in at-risk individuals, such as the household contacts of individuals who display cholera symptoms. By limiting spread within households, the overall burden of the disease could be reduced. The application of phages as prophylactic treatments for mucosal pathogens represents a fast and specific means by which to restrict the impact of bacterial infections on human health.

## Methods

### Growth conditions and strains

Bacterial strains and phages used in this study are listed in Table 1. *V. cholerae* strains were grown in Luria–Bertani (LB) broth supplemented with 100 μg ml^−1^ streptomycin (Sm). The ICP cocktail comprises the *Vibrio* phages ICP1, ICP2 and ICP3 in equal number. These phages were previously isolated from Bangladeshi cholera patient rice-water stool samples^12^. ICP1 was isolated on strain AC4741, whereas ICP2 and ICP3 were isolated on AC53, an Sm-resistant isolate of E7946. All experiments were carried out using AC53 *V. cholerae* O1 El Tor strain E7946. Strains AC4653 and AC2846 were used in plaque assays as negative controls. ICP1 and ICP3 cannot plaque on AC4653, whereas ICP2 cannot plaque on AC2846.

### Bacteriophage preparation

High-titre stocks of the ICP phages were prepared by growth on agar plates followed by polyethylene glycol (PEG) precipitation. Briefly, each phage was grown with the appropriate *V. cholerae* strain in soft agarose (LB broth supplemented with 0.3% agarose) overlays. Once confluent, overlays were incubated with STE buffer (100 mM NaCl, 10 mM Tris and 1 mM EDTA) overnight at 4 °C with gentle rocking to elute phage. The STE-phage solution was clarified by centrifugation, sterile-filtered and incubated with 1 × PEG (4% PEG 8000, 0.5 M NaCl) at 4 °C for 1–3 days, to allow for phage precipitation. Phages were collected by centrifugation at 10,000 *g* for 15 min at 4 °C and the phage pellet re-suspended in STE buffer. Phages were titred by plaque assay, as previously described^10^.

### *In vitro* phage-killing assay

Overnight cultures originating from single colonies of *V. cholerae* were diluted back to an OD_600_ of 0.05 in 50 ml LB supplemented with 100 μg ml^−1^ Sm and grown at 37 °C with aeration. After 15 min, phages were added to each culture. At the indicated time-points, samples were collected to measure (i) CFU per ml and (ii) PFU per ml.

### Infection of infant mice with *V. cholerae*

All animal experiments were in accordance with the rules of the Department of Laboratory Animal Medicine at Tufts University and the Institutional Animal Care and Use Committee. Four- and 5-day-old CD-1 infant mice (Charles River Laboratories) were infected. Each group of mice was comprised of animals from at least two different litters. Both male and female animals were used in this study. There was no observable correlation between sex of the animal and outcome of each experiment. Numbers of mice used in each condition in each experiment are listed in the legends of Figs 2 and 3. These mice were not tested for the presence of resident *V. cholerae* phages as the animals were not previously exposed to *V. cholerae*. As such, we do not predict that they will harbour any *V. cholerae*-specific phages in their microbiome.

Infant mice in phage-treated groups were orogastrically dosed with phage diluted in 2.5% sodium bicarbonate. Infant mice in cholera-treated groups received ca. 10^5^ CFU (‘normal' infectious dose) or ca. 10^8^ CFU (‘high' infectious dose) of *V. cholerae* diluted in 2.5% sodium bicarbonate. As phages were given at least 3 h before bacteria, any multiplicity of infection calculated would be inaccurate. Instead, the titres of bacteria and phage inoculated into animals were calculated from the input materials and the ranges specified in the figure legends. Mice were killed 24 h post infection, the longest infection time permitted in our protocol. Small intestines were dissected and homogenized in LB broth supplemented with 20% glycerol. Cells were serially diluted and plated on LB agar supplemented with 100 μg ml^−1^ Sm and CFU per small intestine was calculated. To extract phage from small intestine homogenates, an aliquot of the intestinal homogenate was treated with chloroform and centrifuged at 10,000 *g* for 5 min. The supernatant was collected and used in plaque assays, to allow for calculation of PFU per small intestine. To assess the resistance profile of *V. cholerae* cells that survive phage prophylaxis, up to ten colonies were randomly picked per animal for use in EOP assays with ICP1, ICP2 and ICP3.

### Infection of infant rabbits with *V. cholerae*

Three-day-old New Zealand White infant rabbits (Charles River Laboratories) were used in this study. Each group of rabbits comprises animals from at least two different litters. Both male and female animals were used in this study. There was no observable correlation between sex of the animal and outcome of each experiment. Infant rabbits in phage-treated groups were orogastrically dosed with phage diluted in 2.5% sodium bicarbonate. Three hours before infection with *V. cholerae*, infant rabbits were pre-treated with ranitidine hydrochloride to reduce stomach acidity (Caraco Pharmaceutical Laboratories) by intraperitoneal injection (2 μg per gram body weight). Infant rabbits were infected with 5 × 10^8^ CFU *V. cholerae* AC53 diluted in 2.5% sodium bicarbonate. As for the mice experiments, exact titres of bacteria and phage inoculated were calculated from the input material and a range is detailed in the figure legends. Animals were weighed at the start of the experiment and periodically throughout the course of the infection. Percentage body weight was calculated by dividing body weight at the end of the infection period by body weight at the start. Animals were killed 12–20 h post infection. Infant rabbits that suffer from cholera typically lose 10–15% of their body weight within 12–14 h and are killed at this time. Infant rabbits that lost <10% of their body weight are killed 20 h post infection, in accordance with our Institutional Animal Care and Use Committee protocol. This time restriction is necessary, as infants cannot be placed back with their mother after *V. cholerae* infection due to culling behaviour and so are without food for the infection period.

After dissection, the intestines of each rabbit were homogenized in LB broth supplemented with 20% glycerol. Caecal fluid, if present, was collected with a 1 ml syringe. Caecal fluid and intestinal homogenates were serially diluted and plated on LB agar containing 100 μg ml^−1^ Sm for calculation of CFU per intestine. Phages were collected from homogenates as described for infection of infant mice. Surviving *V. cholerae* colonies were colony-purified and assessed by EOP assays for resistance to ICP phages.

### EOP assays

EOP assays were performed for isolates that survived phage predation. Each phage was titered on the isolate, on AC53 and also a phage-resistant strain as a negative control (Δ*wbeL* AC4653 for ICP1 and ICP3; Δ*ompU* AC2846 for ICP2). The EOP was calculated by dividing the titre of the phage on the animal isolate by the titre of the phage on AC53. The limit of detection was 1 × 10^-6^. Isolates were described as resistant when the EOP was<1 × 10^−6^, sensitive when the EOP was>1 × 10^−1^ or partially sensitive when the EOP was between these values.

### Sequence analysis of phage-resistant isolates

*V. cholerae* genomic DNA was extracted using a DNeasy Blood & Tissue Kit (Qiagen). Whole-genome libraries were prepared for single-end 150-bp sequencing using the Nextera XT DNA Library Preparation Kit (Illumina). Sequencing was conducted at the Tufts University Core Facility using an Illumina HiSeq 2500. Genomes were assembled using CLC Genomics Workbench 8 software and aligned to the *V. cholerae* O1 N16961 reference genome^13^. To determine the mutations that may confer phage resistance, variant analysis was performed on mapped reads with a frequency threshold of 51%. Results were compared with AC53 variants^14^ to remove those found in the wild-type inoculum. A surviving isolate that was determined to be sensitive to all three ICP phages was sequenced and resulting variants were also removed from resistant isolates variant analyses.

### Data availability

The authors declare that the data supporting the findings of this study are available within the paper and its Supplementary Information files.

## Additional information

**How to cite this article:** Yen, M. *et al*. A cocktail of three virulent bacteriophages prevents *Vibrio cholerae* infection in animal models. *Nat. Commun.* **8,** 14187 doi: 10.1038/ncomms14187 (2017).

**Publisher's note:** Springer Nature remains neutral with regard to jurisdictional claims in published maps and institutional affiliations.

## Supplementary Material

Supplementary Information Supplementary Figure and Supplementary Tables

Supplementary Data 1 Sensitivity of surviving mouse isolates to ICP phages. Refers to data presented in Figure 3 and Supplementary Table 1. Sensitivity was determined by efficiency of plating (EOP).

Supplementary Data 2 Mutations in selected surviving mouse isolates. Selected isolates (from Figure 3, Supplementary Table 1, and Supplementary Data 1) were submitted to whole-genome sequencing, and variant analysis was performed on reads mapped to the *V. cholerae* N16961 genome, chromosomes I and II (NC_002505, NC_002506). Virulence predictions are based on prior Tn-Seq analyses identifying V. cholerae genes needed in the infant mouse model^13^ and the infant rabbit model^18-20^.

Supplementary Data 3 Sensitivity of surviving rabbit isolates to ICP phages. Refers to data presented in Figure 4 and Supplementary Table 3. Sensitivity was determined by efficiency of plating (EOP).

Supplementary Data 4 Mutations in selected surviving rabbit isolates. Selected *V. cholerae* isolates (from Figure 4, Supplementary Table 3, and Supplementary Data 3) were submitted to whole-genome sequencing, and variant analysis was performed on reads mapped to the *V. cholera* N16961 genome, chromosomes I and II (NC_002505, NC_002506). Virulence predictions are based on prior Tn-Seq analyses identifying *V. cholerae* genes needed in the infant mouse model^13^ and the infant rabbit model^18-20^.

## Figures and Tables

**Table 1:** Bacterial strains and bacteriophages used in this study.

**Strain**	**Description**
AC53	*V. cholerae* O1 El Tor Ogawa E7946 (Sm^R^)^23^
AC2846	E7946 Δ*ompU*^24^
AC4653	E7946 Δ*wbeL*^13^
AC4741	*V. cholerae* O1 El Tor Ogawa (Sm^R^), PLE negative^25^
ICP1	ICP1_2011_A; *Myoviridae*^25^
ICP2	ICP2_2004_A; *Podoviridae*^12^
ICP3	ICP3_2007_A; *Podoviridae*^12^

PLE, PICI-like element; Sm^R^, streptomycin resistance.
