# Supplementary material for: A cocktail of three virulent bacteriophages prevents Vibrio cholerae infection in animal models
Source: Nat Commun. 2017 Feb 1;8:14187. doi: 10.1038/ncomms14187 (PMC5296635; doi:10.1038/ncomms14187)
Supplement: Supplementary Information — Supplementary Figure and Supplementary Tables [file ncomms14187-s1.pdf]

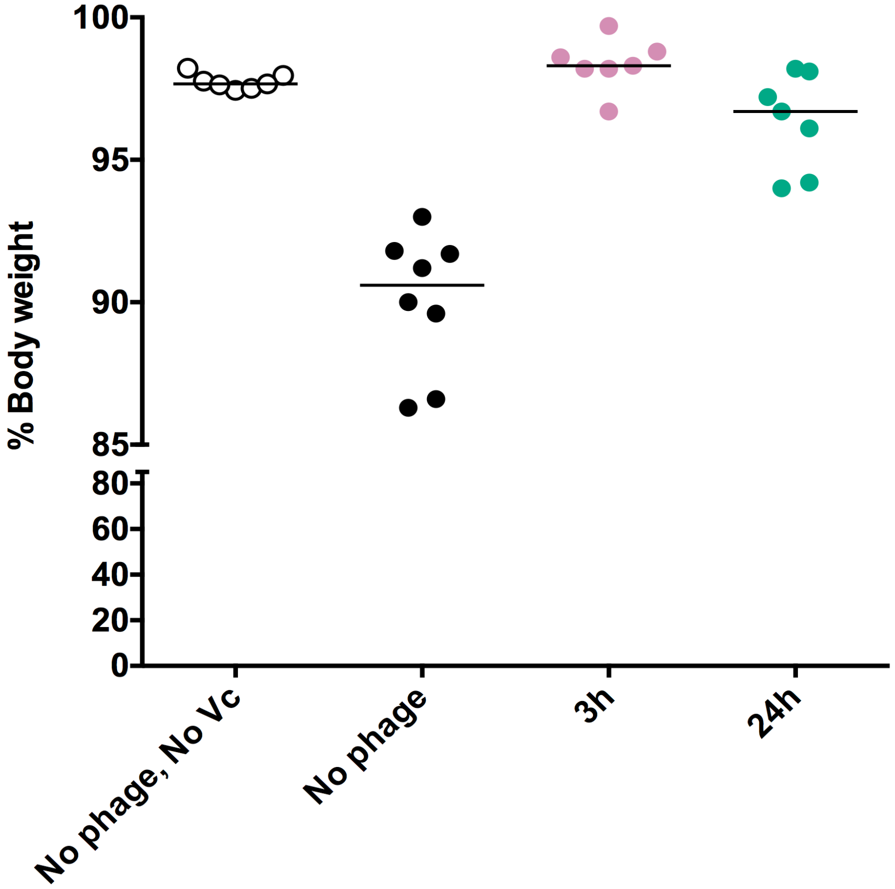

Supplementary Figure 1. Weight loss in infant rabbits infected with *Vibrio cholerae.*
Weight loss in infant rabbits was compared between a group that did not receive phage or *V. cholerae* (no phage, no Vc) and the three groups presented in Figure 4: No-phage, 3-hour phage prophylaxis, and 24-hour phage prophylaxis. Animals were weighed at the start of the infection period and again at the end, and percentage body weight calculated for each animal. The infection period for the no phage group was 12-14 hours and 20 hours for the no treatment and phage-treated groups. Each circle represents one animal. Horizontal lines represent the median.

Resistance Class	Length of Prophylaxis		
	6 h (n=30)	12 h (n=50)	24 h (n=99)
S to all ICP	30 (100%)	50 (100%)	75 (81%)
R to ICP1 only	-	-	-
R to ICP2 only	-	-	14 (14%)
R to ICP3 only	-	-	2 (2%)
PS to ICP2 Only	-	-	4 (4%)
ICP1 (PS) and ICP3 (PS)	-	-	1 (1%)
ICP1 (PS) and ICP3 (R)	-	-	3 (3%)
ICP1 (R) and ICP3 (PS)	-	-	-
R to all ICP	-	-	-

Supplementary Table 1. Phage resistance of *V. cholerae* isolates generated from phage prophylaxis in the infant mouse.
Summary of the phage resistance phenotypes of *V. cholerae* isolates that survived the phage prophylaxis treatment shown in Fig. 2. Resistance was measured by efficiency of plating (EOP) assays. Isolates were described as resistant (R) when EOP $< 1 \times 10^{-6}$, sensitive (S) when EOP $> 1 \times 10^{-1}$, and partially sensitive (PS) when plaques appeared turbid or when the EOP was between 1×10^{-1} and 1×10^{-5}. The data presented refer to the number of isolates displaying a phenotype out of the total number of isolates tested for each time-point. Details of these data can be found in Supplementary Data 1 and 2.

Cecal Fluid Bacterial Counts (CFU/ml)					
No phage		3 hours		24 hours	
Animal 1	6.2×10^8	Animal 9	N/A	Animal 16	5.2×10^5
Animal 2	3.9×10^8	Animal 10	N/A	Animal 17	N/A
Animal 3	8.8×10^8	Animal 11	N/A	Animal 18	N/A
Animal 4	7.8×10^8	Animal 12	N/A	Animal 19	N/A
Animal 5	1.0×10^8	Animal 13	N/A	Animal 20	N/A
Animal 6	1.1×10^9	Animal 14	N/A	Animal 21	N/A
Animal 7	8.2×10^8	Animal 15	N/A	Animal 22	N/A
Animal 8	9.2×10^8			Animal 23	N/A
				Animal 24	N/A
				Animal 25	N/A

Supplementary Table 2. Bacterial counts of collected cecal fluid from phage prophylaxis in the infant rabbit.
Infant rabbits infected with *V. cholerae* develop secretory diarrhea, evidenced by a distended cecum from accumulation of cecal fluid. When possible, cecal fluid was collected from infected animals, and bacteria were enumerated by plating. N/A is used to describe those that did not have cecal fluid, implying that these animals were protected from the onset of cholera symptoms.

Resistance Class	Length of Prophylaxis	
	3 h (n=20)	24 h (n=76)
S to all ICP	4 (20%)	30 (39%)
R to ICP1 only	1 (5%)	2 (3%)
R to ICP2 only	-	-
R to ICP3 only	-	-
ICP1 (PS) and ICP3 (PS)	2 (10%)	2 (3%)
ICP1 (PS) and ICP3 (R)	2 (10%)	-
ICP1 (R) and ICP3 (PS)	3 (15%)	8 (11%)
ICP1 (R) and ICP3 (R)	7 (35%)	30 (39%)
R to all ICP	-	-

Supplementary Table 3. Phage resistance of *V. cholerae* isolates generated from phage prophylaxis in the infant rabbit.
Summary of the phage resistance phenotypes of isolates that survived the phage prophylaxis treatment shown in Fig. 3. Resistance was measured by efficiency of plating (EOP) assays. Isolates were described as being resistant (R) when EOP $< 1 \times 10^{-6}$, sensitive (S) when EOP $> 1 \times 10^{-1}$, and partially sensitive (PS) when plaques appeared turbid or when the EOP was between 1×10^{-1} and 1×10^{-5}. The data presented refer to the number of isolates displaying a phenotype out of the total number of isolates tested for each time-point. Details of these data can be found in Supplementary Data 3 and 4.
